# Structural properties of conductive polymer blends interfaced with water: computational insights from PEDOT:PSS†

**DOI:** 10.1039/d4tc03066d

**Published:** 2024-10-18

**Authors:** Amali G. Guruge, Hesam Makki, Alessandro Troisi

**Affiliations:** a Department of Chemistry, University of Liverpool Liverpool L69 3BX UK amali.galappaththi-guruge@liverpool.ac.uk

## Abstract

In various bioelectronic applications, conductive polymers come into contact with biological tissues, where water is the major component. In this study, we investigated the interface between the conductive polymer poly(3,4-ethylenedioxythiophene):polystyrene sulfonate (PEDOT:PSS) and water, focusing on how the morphology of the PEDOT:PSS is altered by water permeation. We constructed well-equilibrated PEDOT:PSS–water systems in both PEDOT- and PSS-rich phases. Our findings show that water permeates into the polymer through a complex network of water channels, which exhibit a similar pore size distribution in both PEDOT- and PSS-rich phases, leading to similar water intake in these phases. Compared to the dry state of the polymer, water permeation leads to the formation of smaller, less ordered, and distantly located lamella crystallites, potentially resulting in reduced conductivity. Therefore, we argue that these structural changes from the dry state of the polymer to the wet state may be the origin of the significant conductivity reduction observed experimentally in PEDOT:PSS in water or PEDOT:PSS hydrogels.

## Abstract 2

The PEDOT:PSS–water interface was modeled using atomistic MD simulations. The morphological changes in the PEDOT- and PSS-rich phases caused by water permeation were investigated, predicting how these changes influence the material's conductivity.

## Graphical abstract

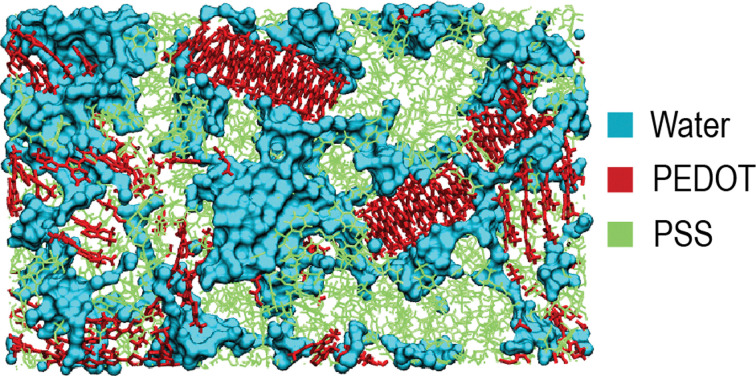

## 1. Introduction

Conductive polymers (CPs) have recently found numerous applications in bioelectronics, where they are interfaced with tissues or fluids to sense, monitor, or stimulate electrical signals from biological systems.^1,2^ In contrast to common materials in bioelectronics, such as silicone and metals,^3^ which have poor flexibility and non-stretchability and are not favorable for integration with biological cells,^4^ CPs, a family of organic polymers, offer flexibility and stretchability. This makes them compatible and desirable for interfacing with biological entities such as tissues, cells, and organs.^5^

**Fig. 1:**
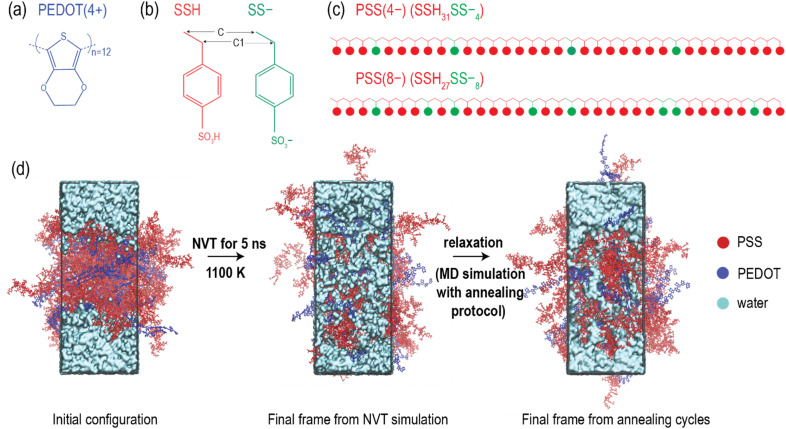
(a) Chemical structure of PEDOT. (b) Chemical structures of repeat units (*i.e.*, SSH and SS-) in PSS. The backbone carbon atoms C and C1 in the PSS chain are illustrated. (c) PSS model used in the current study, consisting of 35 repeat units in total, with 4 or 8 randomly placed SS- monomers and 31 or 27 SSH monomers, respectively, in the PSS chain.^27^ (d) MD simulation steps followed in the study. The initial configuration underwent an NVT simulation at 1100 K for 5 ns, and the final configuration from the NVT simulation was subsequently utilized for a MD simulation with the annealing protocol.

From a chemical perspective, CPs are conjugated polymers with backbones consisting of altering single and double bonds. This arrangement creates delocalized π electrons across the polymer chain, enabling electrical conductivity. One of the most studied and commercially successful CPs used in bioelectronic devices is poly(3,4-ethylenedioxythiophene):polystyrene sulfonate, commonly known as PEDOT:PSS.^6–9^ It is a p-type doped semiconductor material formed from two types of polymers: positively charged short chains containing a few tens of ethylene dioxythiophene units and negatively charged long chains composed of styrene sulfonate units, held together by electrostatic interactions (see Fig. 1(a) for the chemical structure of PEDOT and Fig. 1(b) for the chemical structure of the monomer units of PSS). The morphology of PEDOT:PSS has been studied extensively to understand its polymeric nature.^10–12^ The material's morphology is highly intricate and depends on factors such as PEDOT/PSS composition, synthesis method, post-treatment procedures, solvents, pH, and numerous other variables.^13^ Microscopical investigations carried out by Lang *et al.* and Timpanaro *et al.* revealed that PEDOT:PSS thin films consist of grains with diameters ranging from about 10 to 50 nm, with individual grains exhibiting PEDOT-rich (conductive) and PSS-rich (less conductive) regions with a thickness of 5–10 nm.^11,14^ PEDOT:PSS offers tunable conductivity, modifiable surface structure, ease of preparation and processability, optical transparency, ion and electron transport capabilities, excellent thermal stability, and self-healing properties.^15–18^ Advanced experiments are extensively being conducted to further enhance its mechanical^19–23^ and electronic properties^24–26^ for future utilization.

The applications of PEDOT:PSS are extensive, particularly in biomedical sensors (*i.e.*, biosensors), where the interface between the conductive material and biological substances is pivotal for functionality.^7^ For example, in biosensors, the conductive material in the bioelectronic device interfaces with various biological tissues^28–35^ to transduce biological signals into electrical signals and *vice versa*. As the interface serves as the initial point of contact between biological and electronic materials, gaining an enhanced understanding of the chemical and morphological nature of conductive materials at the interface is essential for advancing the field of bioelectronics.

Water is a major component of the biological environment, and it is experimentally known that PEDOT:PSS swells in water.^36,37^ However, none of the studies have reported the morphological changes observed from the dry state of the polymer (dry film) to the water-permeated polymer (wet film) in terms of the PEDOT lamellae present in PEDOT- and PSS-rich phases, which significantly influence the material's overall electrical conductivity. Thus, the fundamental morphological changes when dry polymer interfaces with water remain poorly understood, although this understanding is essential for advancing this material in bioelectronics. Moreover, due to the complex nature of the conductive material and biological environment, obtaining detailed atomic information on these morphological changes through experimental investigations is challenging. Molecular dynamics (MD) is a complementary tool that enables the exploration of complex systems at the atomic level,^38–40^ facilitating a deep understanding of the system's nature under different conditions. It has already been demonstrated that MD is useful for investigating various aspects of PEDOT:PSS, including its two-phase organization in the dry state^13,27,41^ and in the presence of water,^42^ thermal conductivity,^43^ water intake,^13^ ion diffusion and distribution,^44^ self-assembly on cellulose surface,^45^ the effect of pH^41^ and ionic liquids,^46–49^ and the impact of solvent treatment.^50–53^ Thus, MD serves as an ideal approach to examine the structural changes in PEDOT:PSS (*i.e.*, PSS- and PEDOT-rich) as water permeates into the polymer *via* the CP–water interface at the atomic level. Furthermore, previous studies that modeled PEDOT:PSS in the presence of water^13^ employed coarse-grained (CG) models. While these models are highly effective for generating equilibrated glassy polymer systems due to their faster equilibration, they have certain limitations. Specifically, they tend to provide only qualitative insights into molecular diffusion and often fail to capture critical polymer–water interactions, such as hydrogen bonding, due to their lack of atomistic detail.^54^ In this study, to complement the understanding provided by CG models, we aimed to construct an atomistic model that specifically captures the morphological changes in conductive polymers as they interact with water and water permeates the polymer. Our goal was to determine whether these morphological changes contribute to the sensing mechanism (*e.g.*, changes in electric conductivity) in PEDOT:PSS.

## 2. Methods

We utilized four well-equilibrated initial configurations of PEDOT:PSS in PEDOT- and PSS-rich phases (*i.e.*, PEDOT-rich-I, PEDOT-rich-II, PSS-rich-II, and PSS-rich-I) from a previous study.^27^ These phases represent experimentally obtained PEDOT concentration ranges.^10^ The specific PEDOT concentrations in each structure are detailed in Table 1. It is important to note that these models were robustly equilibrated and accurately reproduced the average lamella crystallite size, characteristic π–π stacking distance, and X-ray diffraction patterns consistent with the experimental results.^27^

**Table 1:** Composition and MD simulation details of the PEDOT-rich and PSS-rich phases

Phase	PEDOT wt% in PEDOT:PSS	PEDOT chains	PSS(−4)	PSS(−8)	No. of water molecules
PEDOT-rich-I	52	120	0	60	15 070
PEDOT-rich-II	43	100	20	40	13 900
PSS-rich-II	35	80	40	20	13 380
PSS-rich-I	26	60	60	0	12 860

In the PSS- and PEDOT-rich phases mentioned above, PEDOT was modeled with 12 repeating units in the bipolaronic state (+4) (see Fig. 1(a)).^27^ PSS was modelled with 35 repeating units, with four or eight SS- monomers [PSS(−4) chain or PSS(−8) chain] randomly placed within the PSS chain (see Fig. 1(b) and (c)),^27^ alongside the SSH monomers. This approach ensured the generation of statistically independent mixture of PSS chains with various repeating unit sequences.^41^ The total charge of the system was preserved by adjusting the correct number of PEDOT chains and PSS chains (*e.g.*, the PSS-rich-I phase contained 60 PSS(−4) chains and 60 PEDOT(+4) chains, see Table 1 for PEDOT/PSS ratio for other phase structures).

### 2.1. Construction of PEDOT:PSS–water interfaces

The construction of the initial interface structures for all four phases began with well-equilibrated PEDOT:PSS models from a previous study.^27^ In the *y*-direction of these equilibrated structures, two layers of water, each with a thickness of 3.0 nm, were applied to both the top and bottom surfaces without breaking any polymer chain. This created vacuum regions within the constructed interface structures, which need to be eliminated (see 2.2 MD simulations).

### 2.2. MD simulations

All simulations were conducted using GROMACS^55,56^ version 2022. Water was modeled using the SPC/E model. For NVT simulations at 1100 K, we used a time step of 1 fs, as larger timestep (*e.g.*, 2 fs) could cause the MD simulation to become unstable due to the high kinetic energy. For MD simulations using the moderate annealing protocol, a time step of 2 fs was employed. PSS was modeled using the Generalized Amber Force Field (GAFF),^57^ while PEDOT was modeled using recently developed and verified all-atom force field parameters,^58,59^ which are compatible with the GAFF. These GAFF compatible parameters were derived from DFT calculations of molecular geometries, vibrational energies, and torsional profiles.^58,59^ They successfully reproduced key morphological parameters such as π–π stacking distance, and the average size of PEDOT lamella crystallites,^27,43^ consistent with experimental results, all of which are related to the charge transfer ability of PEDOT:PSS. This validation supports the use of these forcefield parameters in the current study to predict morphological changes that could impact the material's conductivity. The velocity rescale^60^ algorithm was used for temperature coupling in all simulations. Isotropic pressure coupling was employed with Berendsen^61^ (during the initial equilibration) or Parrinello-Rahman^62^ (in NVT and simulated annealing) algorithms, with a reference pressure of 1 bar and compressibility of 4.5 × 10^−5^ bar^−1^. The Verlet cutoff scheme^63^ with a 1.4 nm cutoff distance was used for short-range non-bonded interactions. For long-range electrostatic interactions, particle-mesh Ewald^64^ (PME) was applied with a grid spacing of 0.12 nm.

We initiated MD simulations to remove the vacuum regions and achieve the correct system volume in the initial interface structures constructed in Section 2.1 of the Methods. To accomplish this, a short MD simulation (*e.g.*, NVT at 298 K) was performed, resulting in the initial configuration shown in Fig. 1(d). This procedure was followed for all four interfaces. Since PEDOT:PSS exhibits a high glass transition temperature (*T*_g_) of approximately 1050 K^65^ in simulations, it is challenging to simulate sufficient water permeation into the polymer at low temperatures using MD simulations within accessible timeframes. Also, the sub-*T*_g_ annealing protocol,^65^ which is conventionally used to equilibrate PEDOT:PSS, cannot be applied to PEDOT:PSS–water systems, as the high temperatures involved in the annealing protocol cause significant volume fluctuations due to super-heated water, disrupting the equilibration of water within the polymer. Therefore, to address the issue of water permeation caused by the high *T*_g_ of PEDOT:PSS, we induced the mixing of water with PEDOT:PSS by conducting an NVT simulation at 1100 K (above the *T*_g_ of PEDOT:PSS) for 5 ns, using the initial configuration shown in Fig. 1(d). This simulation allowed for greater water permeation (approximately 25% of the total, with potential under- or over-soaking) into the glassy polymer. To address the issue of volume fluctuations caused by super-heated water during the annealing process, the final frame from the NVT simulation at 1100 K (see Fig. 1(d)) was subjected to MD simulation with an annealing protocol at moderate temperatures, ranging from 310 K to 360 K over a cycle of 25 ns (see Table S1 in the ESI†). This MD simulation was conducted over 2.4 μs (96 relaxation cycles) to equilibrate the PEDOT:PSS–water systems. The same steps were applied to all four water-permeated structures in the PEDOT- and PSS-rich phases. A schematic diagram of the MD protocol used in this study is shown in Fig. 1(d). It is important to note that this protocol is specifically designed for use with glassy PEDOT:PSS–water systems to achieve sufficient water permeation at room temperature and subsequently equilibrate the water-permeated systems.

### 2.3. Simulation analysis

The following specialized analyses were performed to predict the structural properties of PEDOT:PSS as water permeates into the polymer. Further details on the implementation are provided in the ESI† (Section S1).

I. Lamella crystallite size and number of π–π stacked EDOT pairs –To study the changes in lamella crystallites (a lamella crystallite is a cluster of stacked PEDOT chains that exhibit π–π interactions) due to water permeation into the polymer, we analyzed the average number of PEDOT chains present in a lamella crystallite and the average number of π–π stacked EDOT pairs in each interface structure. (Section S1, I in the ESI†).

II. Orientation parameter of PEDOT – To investigate the changes in PEDOT chain alignment due to water permeation into the polymer, we determined the degree to which individual PEDOT chains were aligned to a defined director (order parameter of PEDOT). (Section S1, II in the ESI†).

III. Connectivity between lamella crystallites – To study the effect of water permeation on the distance between lamella crystallites, we calculated the shortest distances between the sp^2^ carbons in two lamella crystallites (non π–π stack interaction as shown in the illustration in Fig. 4(a)) in each structure by varying the threshold distance from 0.3–1.0 nm. (Section S1, III in the ESI†).

For all the analyses mentioned above, the equilibrated MD trajectories were used. In addition to the analysis methods mentioned above, conventional tools available in GROMACS were employed to calculate the radial distribution functions (RDFs) between oxygen atoms in water and the carbon backbone of PSS (Fig. 1(b)), as well as the root mean square deviations (RMSDs) of the PEDOT and PSS groups relative to the initial structure used in the MD simulation with the annealing protocol. Where applicable, the computed structural parameters in the wet film were compared with those in the dry film of PEDOT:PSS. Frames from the MD simulations were visually examined using VMD.^66^

Apart from the above analyses, the weight percentage of water to polymer was calculated in the bulk polymer region of each phase (detailed information on this calculation is included in Section S1 of the ESI†). The Largest cavity diameter (LCD), pore limiting diameter (PLD), and pore size distribution (PSD) were calculated to characterize the pore morphology in both wet and dry films. The PLD, LCD, and PSD calculations were performed using the PoreBlazer^67^ tool. For these calculations in the wet film, we selected a boxed region with the maximum possible size in the water channel area of each phase structure, avoiding the bulk water region at the interface. For the dry film calculations, a region of similar size to the wet film calculation in each phase was selected. To detect the geometry of the pores in water, we defined the PLD based on a water model diameter of 0.31 nm.^68^

The final frames obtained after 2.4 μs (96 relaxation cycles) for the water-penetrated PEDOT- and PSS-rich structures, along with the forcefield parameters and input files used in the MD simulations are available in a public repository: https://github.com/HMakkiMD/PEDOT-PSS/tree/main/GROMACS-files/Wet-Films.

## 3. Results and discussion

To make reliable predictions of structural changes in the conductive polymer due to water permeation, it is essential to ensure that the MD systems reach equilibration. We took several steps within our MD protocol to verify the equilibration of the models. First, we examined two morphological parameters: the number of lamella crystallites and the average size of the lamella crystallites in each structure, which are expected to fluctuate until the systems reach equilibrium. We observed that these properties fluctuated over time and became relatively constant with further annealing cycles (Fig. S2(a) and (b), ESI† yellow shaded area indicates the equilibrated region). Secondly, we analysed the interactions between water and PSS by calculating the RDFs between the water oxygen atoms (OW) and the backbone carbon atoms of PSS (C and C1 in Fig. 1(b)). We found that the RDFs varied with the number of relaxation cycles but remained stable during the final five cycles of the simulation, as shown in Fig. S2(c) in the ESI.† Thirdly, we traced the trajectory of the water molecules during the last 10 ns of the simulation to examine their motion. We observed that water molecules were exchanged between the channel and the bulk water region, as shown in Fig. S2(d) in the ESI.† This movement further indicated that the water molecules inside the polymer were not trapped in a metastable state. Lastly, for the PEDOT-rich-I and PSS-rich-I phases, we computed the RMSDs for the PEDOT and PSS groups relative to the initial configuration used in the MD simulation with the annealing protocol, as shown in Fig. S3(a) (ESI†). The results indicated significant mobility of the PEDOT and PSS chains during the equilibration process, ensuring sufficient polymer mobility. The RMSD reached a maximum of approximately 7–8 nm for both PEDOT and PSS in the PEDOT-rich-I and PSS-rich-I phases, which is more than twice the radius of gyration for PEDOT (∼1.3 nm) and PSS (∼1.8 nm) in the dry film,^27^ further confirming the polymer's sufficient mobility to reach equilibration. Additionally, Fig. S3(a) in the ESI† indicates that after the 24th cycle, the RMSD fluctuates around a stable value. This fluctuation suggests that while the molecules remain mobile, they maintain their structural conformations. Fig. S3(b) in the ESI† supports this observation, as we noted that while the PEDOT and PSS chains moved slightly after the 24th cycle with minimal conformational changes, they exhibited significant movement before the 24th cycle, accompanied by noticeable conformational changes. All the outcomes from the above analyses, such as achieving stable fluctuation in the morphological parameters and RMSDs of PEDOT and PSS, along with stable RDFs during the final annealing cycles and the movement of water molecules between the channel and bulk water regions, support the successful generation of well-equilibrated PEDOT:PSS–water models using the MD protocol in this study.

Using the equilibrated models, we first present the structural changes in the PEDOT network, focusing on lamella crystallite size, the number π–π stacked pairs, order parameter, and inter-lamella connectivity observed in the wet film of PEDOT:PSS compared to the dry film. These structural parameters are related to the conductivity of PEDOT:PSS,^69–72^ and we discuss how they are connected to conductivity in Sections 3.2 and 3.3. Next, we study and characterize the water channel size in PEDOT- and PSS-rich phases with varying PEDOT concentrations. Finally, we conclude by discussing how the properties analyzed in this study impact the conductivity of wet PEDOT:PSS.

### 3.1. PEDOT network in the interfaced structures

**Fig. 2:**
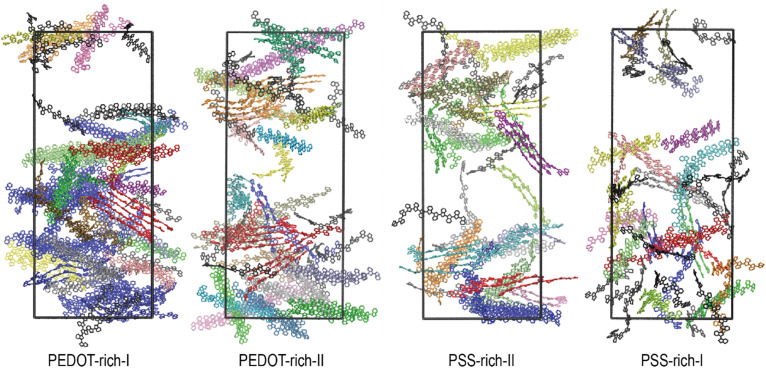
Side view of the final frames of each phase after 96 relaxation cycles. Each colour represents an individual lamella crystallite, with isolated PEDOT chains shown in black across all phases. PSS chains and water were omitted for clarity.

We first report how the formation of lamellae is influenced by the process of wetting as both PEDOT-rich and PSS-rich phases in the dry film consist of PEDOT lamellae embedded in PSS chains.^27^ For this purpose, we first visually inspected the arrangement of PEDOT lamella crystallites in wet PEDOT- and PSS-rich phases, as shown in Fig. 2. We observed the formation of small lamella crystallites in the wet film compared to the dry film data reported in the literature (see Section 3.2, lamella crystallite size for more information).^27^ On the other hand, we noticed that the highly ordered arrangement of PEDOT in the dry PEDOT-rich-I phase, as reported in the literature, was disrupted by water permeation, while a moderately ordered arrangement was maintained in phases with high PEDOT concentrations. An isotropic PEDOT network was visible in PSS-rich-I phase (see Section 3.2, order parameter for more information). Additionally, within a lamella crystallite, we observed more aligned PEDOT chains in all four phases (see Section 3.2, number of π–π stacked pairs for detailed information).

### 3.2. Average lamella crystallite size, number of π–π stacked pairs, and order parameter

**Fig. 3:**
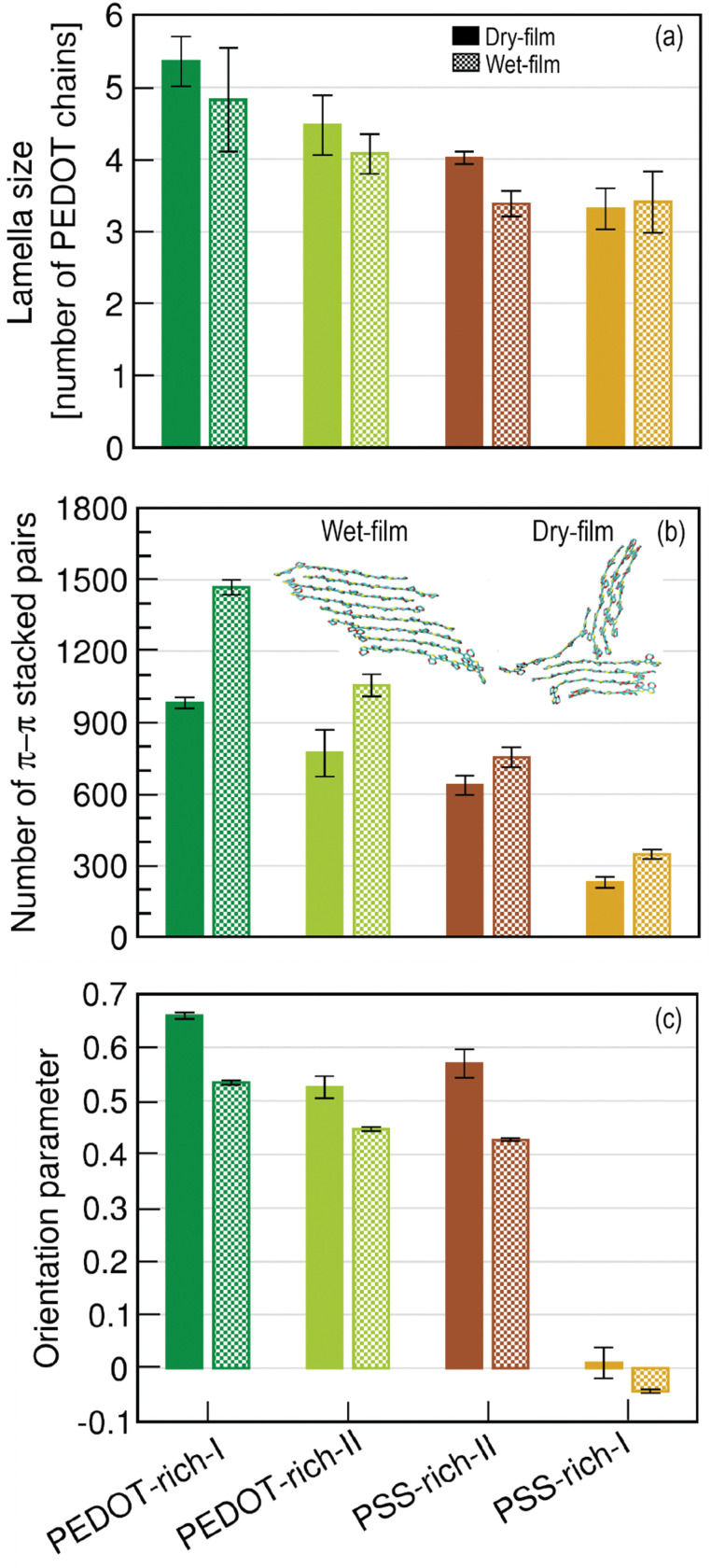
(a) The average lamella crystallite sizes for all phase structures in dry and wet films. (b) The average number of π–π stacked pairs in all phases in dry and wet films. (c) Orientation parameters for all phase structures in dry and wet films. An example of PEDOT chain alignment within a lamella crystallite of size eight in the dry and wet films (cyan, yellow and red represent carbon, sulphur and oxygen atoms respectively) is also shown. The error bars in (a), (b), and (c) of the wet film represent the standard deviation of the lamella crystallite size, π–π stacked pairs, and orientation parameter during the last 15 relaxation cycles. The error bars of the dry film represent the standard deviation of the data during the five last relaxation cycles.

As we visually observed a reduction in the PEDOT lamella crystallite size upon wetting, which affects the material's conductivity, we next investigated this phenomenon quantitatively. Using the last 15 relaxation cycles, we calculated the average lamella crystallite size in each phase of the wet film. Data for the dry and wet films are shown in Fig. 3(a). The average lamella crystallite size (*i.e.*, for PEDOT-rich) obtained from our models was in the same range as those predicted by a computational study^27^ and Volkov *et al.*'s Grazing-Incidence Wide-Angle X-ray Scattering (GIWAXS)^73^ investigations, indicating that our predictions are consistent with previously reported observations. Our data also showed that the average lamella crystallite size in the wet film was further reduced compared to the dry film. A noticeable change in the size of the crystallites was observed in phases with higher PEDOT concentrations (*i.e.*, 53% wt, 43% wt and 35% wt). Generally, charge transport is expected to be more efficient *via* large lamella crystallites.^69^ Thus, the reduction in the average lamella size as water permeates into the polymer decreases the charge transfer ability of the polymer, which is likely responsible for the significant drop in conductivity of PEDOT:PSS in water/hydrogels (*e.g.*, higher than 4000 S cm^−1^ (Kim *et al.*^74^) to ∼20–40 S cm^−1^ (Lu *et al.*^75^)). Additionally, Makki and Troisi,^27^ predicted that the average lamella crystallite size decreases with decreasing concentration of PEDOT in the dry phases (PEDOT concentration varies as PEDOT-rich-I > PEDOT-rich-II > PSS-rich-I > PSS-rich-I). We observed a similar trend as water permeated into the polymer; however, this was not noticeable in the PSS-rich phases. This suggests that water permeation breaks down large lamella crystallite networks in PEDOT-rich phases into smaller lamella crystallites, while this effect is not significant in PSS-rich phases.

It is also known that the relative stacking between polymer chains and the degree of order of crystallites are linked to the carrier mobility (*i.e.*, how fast electrons or holes move) of the material.^70^ Thus, we next investigated the average number of π–π stacked pairs over the last 15 cycles, as illustrated in Fig. 3(b), along with comparable data for the dry film. Across each phase, the average number of π–π stacked pairs was much higher than those in the dry film of the polymer, with a significant increase observed when PEDOT concentration was high (*i.e.*, PEDOT-rich phases). Since we observed a decrease in the average lamella crystallite size (discussed above) in almost all phases of the wet film (except PSS-rich-I), we conclude that the increase in π–π stacked pairs in both PEDOT- and PSS-rich phases is associated with a greater alignment of PEDOT chains within crystallites compared to the polymer in the dry state. This implies the formation of highly ordered PEDOT chains within a lamella crystallite as water permeates into the polymer, compared to the dry polymer. Furthermore, the visual observation further confirmed this phenomenon. An example of PEDOT chain alignment in a lamella crystallite of size eight in dry and wet films is shown in Fig. 3(b), clearly illustrating that the PEDOT chains are more aligned in the crystallite in the wet film. This is consistent with a previous investigation by Kim *et al.*, based on X-ray diffraction and reflectance spectrum studies,^71^ which showed that the addition of a polar solvent (*i.e.*, dimethyl sulfoxide) increases interchain packing and enhances the π–π coupling of PEDOTs, thereby increasing the conductivity of PEDOT:PSS. Furthermore, our data showed that the number of π–π stacked pairs increases with the PEDOT concentration, as observed in the dry film.^27^

Next, we examined the influence of water permeation on the orientation of the PEDOT chains, as well-ordered PEDOT chains enhance the overall conductivity of the material by improving π–π stacking.^71^ The orientation of PEDOT chains along a director (*i.e.*, order parameter *S*) in wet films compared to dry films, indicates a further reduction in *S* in the wet film, as illustrated in Fig. 3(c). This implies that as water permeates into the polymer, the PEDOT chains become less ordered than in the dry film. A notable transition from moderately ordered phases in PEDOT-rich-I, PEDOT-rich-II, and PSS-rich-II to a completely anisotropic phase occurred in the PSS-rich-I phase (Fig. 2), similar to the observation reported in the dry film.^27^ Even though the highly ordered arrangement of PEDOT chains within a lamella crystallite favors charge transfer along the chains, the less ordered PEDOT chains among the lamella crystallites are likely to reduce the overall charge transfer ability of the material. This is due to the reduced possibility of π–π stacking interactions as *S* decreases.

In summary, the analysis of lamella crystallites in the wet film showed that the average lamella crystallite size was further decreased compared to the dry film, which could decrease the conductivity of PEDOT:PSS. A noticeable change in crystallite size was observed at high PEDOT concentrations, indicating the breakdown of larger crystallites into smaller ones due to water permeation. Furthermore, water permeation into the polymer formed more aligned PEDOT chains within a lamella crystallite, which may favour interchain charge transfer ability. However, the presence of less ordered PEDOT chains along the grain director (*i.e.*, reduced *S* in wet films) is likely to decrease the overall conductivity of the polymer. This is due to fewer π–π stacking interactions occurring when PEDOT chains are less ordered among the crystallites (or along the grain director).

### 3.3. Inter-lamella connectivity

**Fig. 4:**
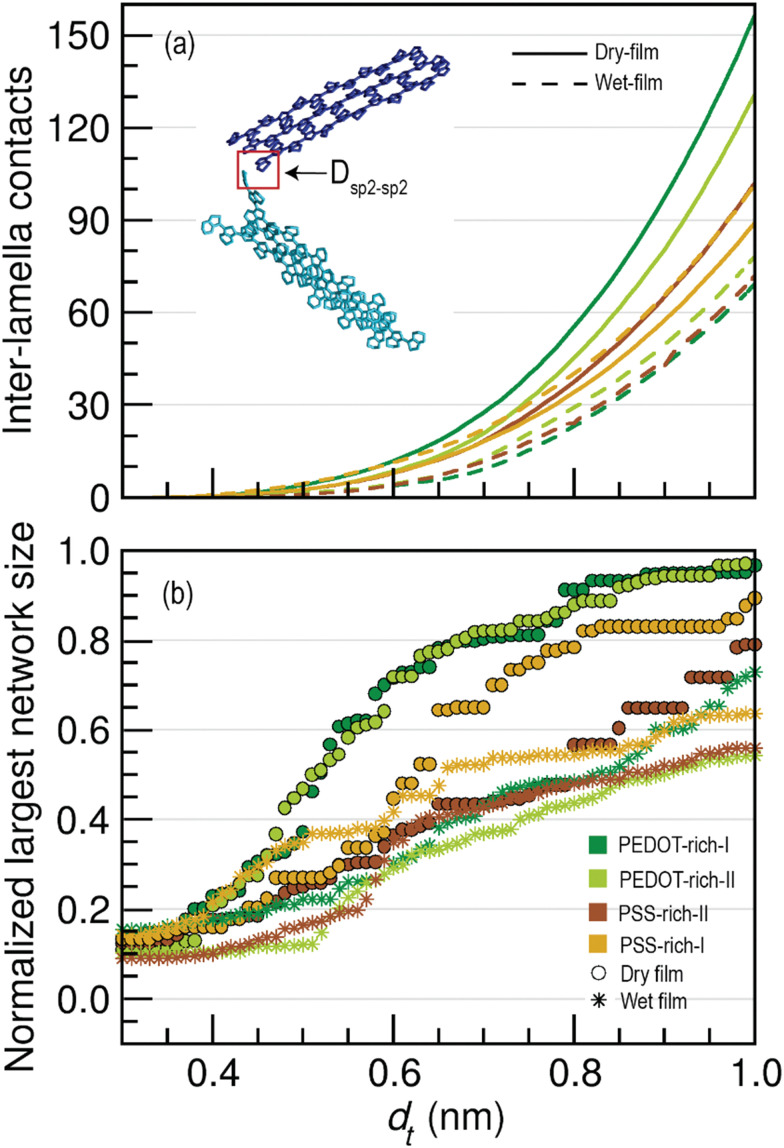
(a) The number of inter-lamella contacts (non π–π stacking) in wet and dry films normalized by the total number of lamellae as a function of *d*_t_. An example of a non π–π stacking contact between two PEDOT lamellae (blue and cyan) is shown in the panel, where *D*_sp^2^–sp^2^_ indicates the distance between one sp^2^ carbon atom in the blue lamella and one sp^2^ carbon atom in the cyan lamella. (b) The normalized largest network size in PEDOT- and PSS-rich phases of wet and dry films as a function of *d*_t_.

In the conductive polymer PEDOT:PSS, having shorter inter-lamella stacking distances enhances the electrical conductivity.^72^ Since there were no entirely π–π stacked connected PEDOT chains in the wet films of PEDOT- and PSS-rich phases in our models, charge transport or electron conduction should occur through the non π–π interactions of disjoint lamella crystallites. In these interactions, the sp^2^ carbons belonging to two lamellae can come close to each other without engaging π–π stack interactions (see the illustration of the blue and cyan crystallites in Fig. 4(a)).

Given the importance of non π–π interactions in the conductivity of the polymer, we investigated the changes in connectivity between disjoint lamella crystallites (including single PEDOT chains) from the dry film to the wet film by computing the number of inter-lamella contacts for a range of distance thresholds (*d*_t_) for *D*_sp^2^–sp^2^_ (0.3–1.0 nm). Through this, we aimed to explore the possible long range tunneling effects between PEDOT chains. The calculated average number of inter-lamella contacts as a function of *d*_t_ for wet and dry films is shown in Fig. 4(a). Note that the total number of contacts in each phase was divided by four (as PEDOT has four sp^2^ carbon atoms) and by the number of lamella crystallites, including the individual chains present in the phase, during the normalization. We observed that non π–π contacts (inter-lamella contacts) increase as *d*_t_ increases, as more crystallites become connected with increasing *d*_t_. However, a significant reduction in inter-lamella contacts was visible in all phases compared to the dry film, highlighting that lamella crystallites in the wet film were not as closely connected as those in the dry film. Furthermore, the reduction in the inter-lamella contacts was more pronounced in the PEDOT-rich phases, indicating that water permeation causes greater segregation of conductive grains in PEDOT-rich phases than in PSS-rich phases. The increased inter-lamella distances in both PEDOT- and PSS-rich phases in wet films may cause an increase in inter-grain spacing, requiring electrons to tunnel over considerably longer distances in wet films compared to the dry film. As better inter-grain conductivity is expected with shorter inter-lamella distances,^27^ having more inter-lamella distances as water permeates is one likely reason for the low conductivity of PEDOT:PSS in water (in hydrogels) that we experimentally observed.^75^

Additionally, we explored the changes in the size of the largest lamella network in PEDOT- and PSS-rich phases of the wet film. Fig. 4(b) illustrates the normalized largest network size in wet and dry films as a function of the threshold distance *d*_t_, which is useful for studying the connectivity of crystallites and for revealing the overall conductivity of the wet film. As expected, more lamella crystallites became connected as the threshold distance increased, thereby increasing the size of the largest crystallite network with *d*_t_. However, the normalized size of the largest network was reduced in the wet film compared to the dry film, with a significant reduction in PEDOT-rich phases. Thus, analysis of the normalized largest network size as a function of *d*_t_ suggested that water permeation causes the formation of distantly spaced crystallites, a phenomenon pronounced at high PEDOT concentrations. Although the normalized size of the largest crystalline network decreased with decreasing PEDOT concentration in the dry film, no significant difference was observed in the wet film. This indicates that the effect of water permeation in PEDOT-rich phases is extensive, resulting in connectivity of conductive grains similar to that observed in wet film of PSS-rich phases. On the other hand, when *d*_t_ was increased from 0 to 0.40 nm, the increase in the normalized largest network size was minimal. However, a significant increase was observed when *d*_t_ exceeded 0.40 nm. This further confirmed that at smaller *d*_t_, the majority of PEDOT lamellar crystallites were not connected in the wet film. This hinders the inter-grain conductivity or pathways that connect conductive regions, causing a reduction in the charge transfer ability of the polymer since the electron tunneling probability decreases by approximately one order of magnitude for every additional 0.1 nm.^76^

In summary, lamella crystallites in the wet film were less well-connected than those in the dry film, with a significant increase in inter-lamella distances observed in phases with high PEDOT concentrations (*i.e.*, PEDOT-rich phases). Consequently, the distance that charge carriers must cover to hop between two lamellae is larger in wet films, resulting in less efficient charge transport.

### 3.4. Water intake and water channels in PEDOT- and PSS-rich phases

**Fig. 5:**
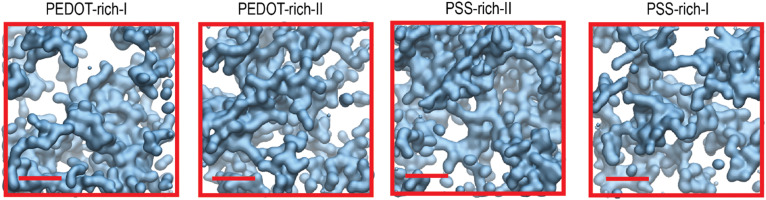
(a) Surface representation of VMD^66^ in water channels inside the cross-section of a 4 nm cubic box in PEDOT- and PSS-rich phases. The scale bar length is 1 nm.

To observe how water disperses inside the polymer, we first visually examined the water channels in the PEDOT- and PSS-rich phases. Fig. 5 illustrates the water channels in a 4 nm cubic box cross-section inside the polymer. Consistent with the observations by Sedghamiz *et al.*,^44^ who reported the presence of water channels when dry PEDOT:PSS was immersed in water, we also observed water channels inside the wet film. Visual observation revealed a connected, complex network of water channels inside the polymer in both PEDOT- and PSS-rich phases, with no noticeable visual differences between them.

We quantitatively investigated the water content in both hydrated PEDOT- and PSS-rich phases by calculating the weight percentage (wt%) of water to polymer in the bulk polymer region (see Section S3 in the ESI†). We found that the weight percentage of water to polymer in the PEDOT-rich-I, PEDOT-rich-II, PSS-rich-II, and PSS-rich-I phases varies as 18.26, 20.06, 21.67, and 17.95, respectively (see Fig. S4 in the ESI†). This indicates that there is no significant difference in the weight ratio of water to polymer between the PEDOT- and PSS-rich phases, implying that water intake does not depend on the phase structure or PEDOT concentration. Our findings differ from the water intake patterns of PEDOT- and PSS-rich phases reported by Modarresi *et al.*^13^ using Martini CG models. This divergence likely arises from the inherent differences in how forcefields are parameterized in atomistic *versus* CG approaches. While both methodologies have been extensively validated (including the forcefield parameters used in our atomistic models^57–59^ as well as those of the Martini model^41,77,78^), each has its own strengths and limitations. In CG models, such as Martini, the selection of bead types plays a critical role in determining the strength of intermolecular interactions.^79^ This means that even slight variations in bead selection can lead to different predictions, such as water intake in PEDOT- and PSS-rich phases. In contrast, atomistic models offer a more detailed representation of molecular interactions, such as hydrogen bonding, leading to a more accurate depiction of water morphology within the polymer. However, our atomistic model predicts a water intake of about 20%, which is lower than the experimental maximum of 56% at 80% relative humidity and 26 °C.^80^ This discrepancy may possibly be due to the drastically shorter PSS chains that atomistic models can accommodate compared to CG models. Thus, by integrating the findings from both methodologies, we can move closer to a more comprehensive understanding of the hydrated PEDOT:PSS morphology.

**Fig. 6:**
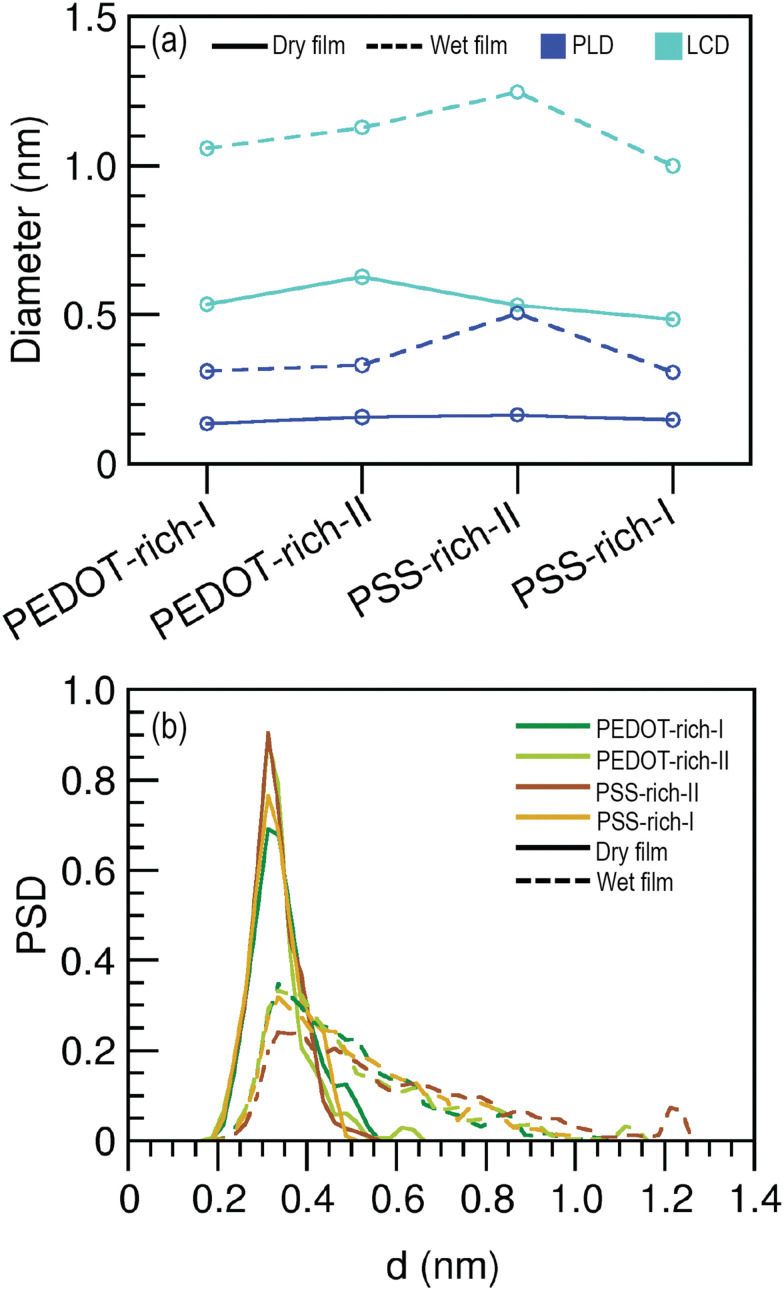
(a) Pore limiting diameter (PLD) and large cavity diameter (LCD) as a function of the phase structures. (b) Pore size distribution (PSD) of PEDOT- and PSS-rich phases as a function of channel width.

As we observed similar water intake regardless of the phase structure, we next investigated the morphology of water channels in PEDOT- and PSS-rich phases in dry and wet films by calculating the PLD and LCD. PLD is the critical diameter for water permeation through a bottleneck in the water channel, while LCD is the maximum pore size in a heterogeneous polymer network.^68^ The calculated PLD and LCD for different phase structures in dry and wet films are shown in Fig. 6(a). Our data indicated that there was no significant difference in PLD or LCD between the PEDOT-rich and PSS-rich phases in dry and wet films. However, both PLD and LCD were higher in wet films than in dry films. A PLD larger than the critical diameter of water (0.31 nm) allows water molecules to move freely through all pores in the structure.^68^ In wet films, the PLD was very similar to the critical diameter of water, suggesting that water molecules can move considerably through all the pores of phase structure. In dry films, the PLD was much smaller than the critical diameter of water, while the LCD was slightly larger, signifying the tight packing of the polymer morphology in the dry state.

To study the overall pore network inside the polymer, we then analyzed the pore size distribution (PSD) in wet and dry films as shown in Fig. 6(b). Data for the dry film indicates that there is no difference in pore sizes between the PEDOT- and PSS-rich phases, and most of the pores inside these phase structures are around 0.3 nm. This implies that pore formation in the dry film is not affected by the PEDOT concentration. The high-intensity peaks for the PSDs with small pore size diameters further confirms the tight packing of the dry polymer in both PEDOT- and PSS-rich phases. However, water permeation into the polymer leads to a broad pore size distribution, with no significant difference between the PEDOT- and PSS-rich phases, suggesting that the pore size in wet films is not influenced by the PEDOT concentration. This explains the similar water intake by the PEDOT- and PSS-rich phases that we previously observed. Sedghamiz *et al.*^44^ argued, based on an MD investigation, that PEDOT:PSS must contain a network of pores that form during the drying process, and these pores are filled with water once the film is immersed in water. Our data suggests the presence of very small pores inside the dry films of PEDOT:PSS. However, as PSD widens due to water permeation, we argue that this process may expand the existing pores in the dry film, resulting in larger diameter pores. Furthermore, the existence of larger pores in our models suggests that increased water intake is possible through the formation of wider water channels and the potential existence of water microdomains within the film. This ultimately causes morphological changes (as we discussed in Sections 3.2 and 3.3), leading to a significant reduction in conductivity, which we observe experimentally.^75^

In summary, the pore characteristics (*i.e.*, PSD, LCD and PSD) were not affected by the PEDOT concentration or phase structure in both the dry and wet films. This explains the similar water intake observed in all phase structures of the wet film. Water permeation may cause an expansion of existing pores in the dry film, leading to larger pore diameters in the wet film compared to the dry film.

## 4. Conclusions

We developed well-equilibrated atomistic models for PEDOT:PSS–water interfaces and simulated water permeation into PEDOT:PSS using molecular dynamics. To the best of our knowledge, this is the first study to use atomistic models to describe the morphological changes in PEDOT:PSS as it interfaces with water and after water penetrates the polymer through the interface.

Our models show that, once PEDOT:PSS interfaces with water, permeation occurs through a connected and complex network of water channels. These channels are similar in size for PEDOT- and PSS-rich phases, leading to a relatively uniform water intake in the material. As claimed by Sedghamiz *et al.*,^44^ these channels could be pre-formed pores in PEDOT:PSS, where water fills in once the material is immersed in water. Our findings further suggest that water permeation subsequently causes an expansion of these dry film pores, resulting in a wider range of pore sizes in the wet film. Water permeation into the polymer leads to the formation of relatively smaller and less ordered lamella crystallites compared to the dry film. Furthermore, within a lamella crystallite, water permeation causes PEDOT chains to become more ordered and better align with each other than in dry film. Our data also suggest that the inter-lamella distance between crystallites is considerably higher in the wet film. We argue that the formation of small, less ordered crystallites, which are distantly located from each other in wet PEDOT:PSS, contributes to the reduction in electrical conductivity observed experimentally^75^ (*e.g.*, PEDOT:PSS in water or PEDOT:PSS hydrogels). As this study provides insight into how conductivity is reduced in wet PEDOT:PSS, the findings could potentially inform the design of biosensors that reliably operate in wet biological environments, such as blood or other body fluids. Additionally, this study serves as a benchmark for modelling PEDOT:PSS–water systems at the atomic level, and we believe that our atomistic models could help refine CG forcefield parameters in the future.

## Data availability

The authors declare that all data supporting the results reported in this study are available within the paper and the ESI.†

## Conflicts of interest

There are no conflicts of interest to declare.

## Supplementary Material

TC-012-D4TC03066D-s001
