# Supplementary material for: Structural properties of conductive polymer blends interfaced with water: computational insights from PEDOT:PSS
Source: J Mater Chem C Mater. 2024 Oct 18;12(47):19245–57. doi: 10.1039/d4tc03066d (PMC11497116; doi:10.1039/d4tc03066d)
Supplement: TC-012-D4TC03066D-s001 [file TC-012-D4TC03066D-s001.pdf]

Supplementary Information (SI) for Journal of Materials Chemistry C.
This journal is © The Royal Society of Chemistry 2024

Structural properties of conductive polymer blends interfaced with water: Computational insights from PEDOT:PSS

Amali G. Guruge*[a], Hesam Makki[a] and Alessandro Troisi[a]

[a]Department of Chemistry, University of Liverpool, Liverpool L69 3BX, UK.

Email: amali.galappaththi-guruge@liverpool.ac.uk

S1- Simulation analysis

Sections I to III provide details on the implementation of the analysis of the morphological properties calculated in this study.

I. Lamella crystallite size and number of π-π stacked EDOT pairs – This was computed using the *pistacking.py* script (*https://github.com/HMakkiMD/PEDOT-PSS/blob/main/Pistacking-git/pistacking.py*).[1] The script defines the plane spanned by the heavy atoms of each EDOT monomer and identifies π-π stacked EDOTs on different chains that satisfy three conditions[1]; i) the angle between the normal vectors of the two EDOT planes is smaller than 10°, ii) the π-π stacking distance ($D_{\pi\text{-}\pi}$ shown in Figure S1(a)) is less than 0.4 nm, and iii) the horizontal distance between the centers of geometry of each parallel pair is less than 0.5 nm. The script also accounts for PEDOT chains that share at least one π-π stacked EDOT pair belonging to a single PEDOT lamella crystallite, enabling the identification of the PEDOT chains per lamella crystallite (lamella crystallite size) and the total number of lamella crystallites present in each phase.

II. Orientation parameter of PEDOT – The orientation of PEDOT chains in four interface structures was studied by calculating the order parameter *S* (see equation 1), which is typically used to evaluate the ordering of the molecules along a director (Figure S1(b)) in liquid crystals[2].

$$S = \left\langle \frac{3\cos^2\theta - 1}{2} \right\rangle \qquad \text{Equation 1}$$

In the above equation, θ is the angle between the PEDOT molecular axis and the grain director (see Figure S1(b)), and the brackets represent the spatial and time average. This calculation was performed using the *orientationparameter.py* script from a previous study (*https://github.com/HMakkiMD/PEDOT-PSS/blob/main/OrientationParameter-git/orientationparameter.py*)[1].

III. Connectivity between lamella crystallites – This was investigated using the *Inter-lamellae.py* script (*https://github.com/HMakkiMD/PEDOT-PSS/blob/main/Inter-lamellae-contact-git/Inter-lamellae.py*) from a previous study.[1] This script calculates the shortest distance between sp^2 carbon atoms ($D_{sp2\text{-}sp2}$) belonging to two lamella crystallites (see the illustration in Figure 4(a)). Furthermore, the script analyses the connectivity of all lamella crystallites

and individual PEDOT chains by varying the shortest distance in the threshold range of 0.3-1.0 nm.[1]

The calculation of the weight percentage of water to polymer inside the bulk polymer region is not straightforward. Therefore, we first plotted the density profiles of water and PEDOT:PSS using *gmx_density* available in GROMACS and then integrated the appropriate areas under the two curves separately. For example, to determine the weight of water, the blue shaded areas in Figure S1(c) were considered, while the areas covered by the PEDOT:PSS density line within the same *x*-coordinates (Figure S1(c), shaded in magenta) were used for the polymer. This integration was performed using a Python script, and the weight percentage was then calculated based on the obtained weights.

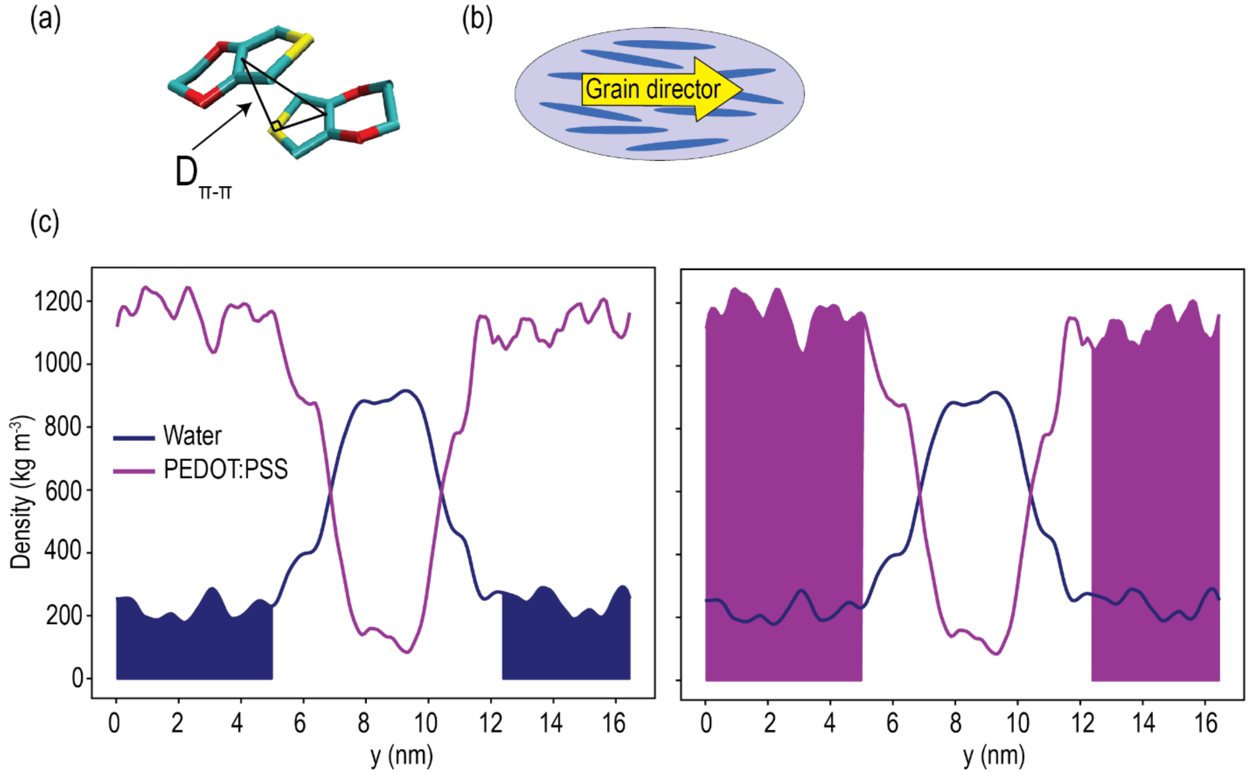

Figure S1 – (a) An example of a π-π stacked EDOT pair. $D_{\pi\text{-}\pi}$ represents the π-π stacking distance in the π-π stacking interaction. (b) Schematic representation of the PEDOT chain orientation (blue ellipses represent PEDOT chains). The yellow arrow indicates the grain director used in the order parameter calculation. (c) Density graphs for water (blue) and PEDOT:PSS (magenta), with integrated regions shaded for water (in blue) and PEDOT:PSS (in magenta) to calculate the weight percentage of water to polymer inside the bulk polymer region.

S2- Reaching equilibration

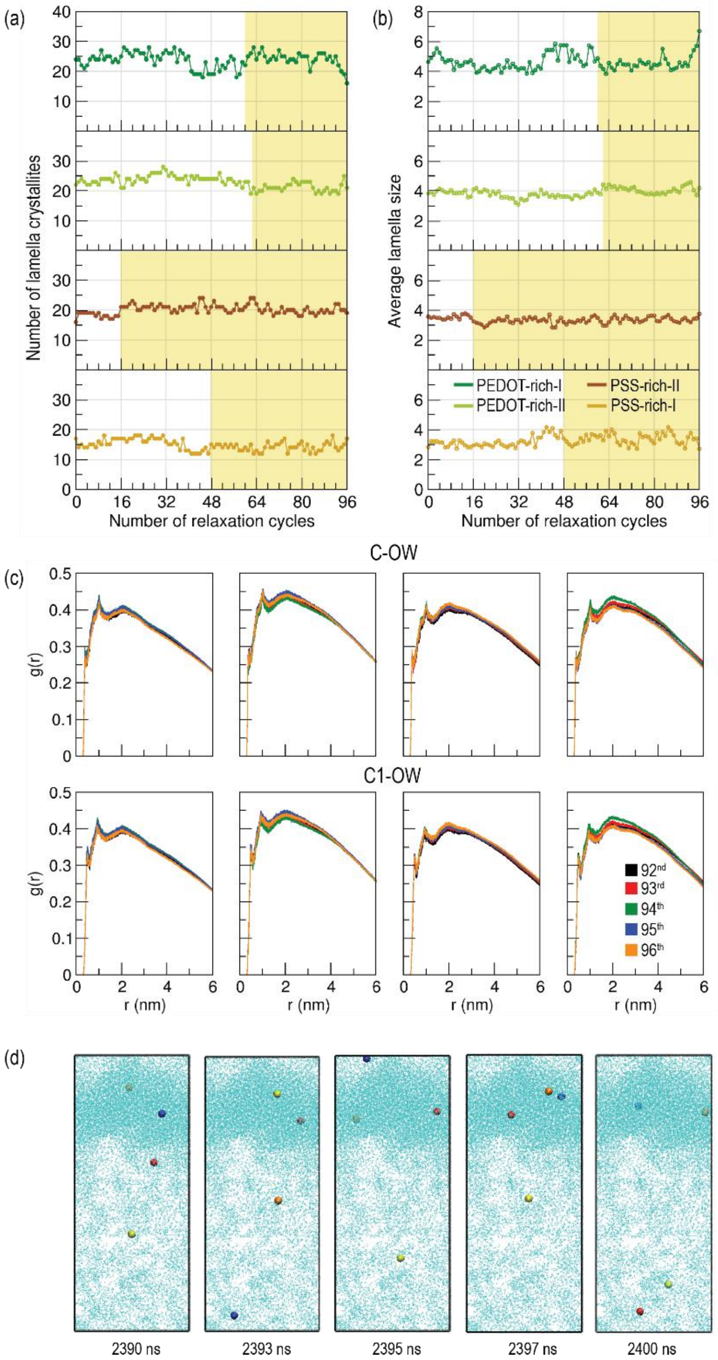

Figure S2 – (a) Number of lamella crystallites for four phases as a function of the number of relaxation cycles. (b) Average lamella crystallite size for four phases as a function of the number of relaxation cycles. The yellow shaded area indicates the equilibrated region. (c) Radial distribution functions (RDFs) computed between backbone C atoms (i.e., C and C1, see Figure 1) of PSS and oxygen atoms in water (OW) at the end of the 92nd, 93rd, 94th, 95th and 96th annealing cycles (using the last 1 ns of each cycle). (d) Snapshots taken during the last 10 ns of the simulation,

showing the movement of four water molecules in an equilibrated system. Water molecules in the bulk water and water channel region are shown in cyan. The van der Waals representation in VMD[3] of the water molecules illustrates their movements: travelling from the bulk region to the channel region and back (water in blue and orange), and moving from the channel region to the bulk region and back (water in red and yellow). PEDOT and PSS have been omitted for clarity.

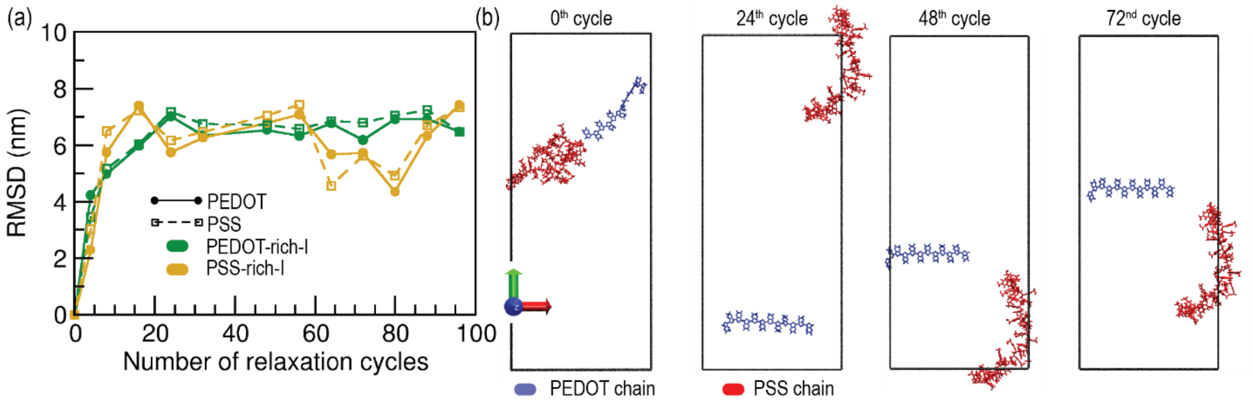

Figure S3 – (a) RMSD values for PEDOT and PSS in the PEDOT-rich-I and PSS-rich-I phases as a function of the number of relaxation cycles. (b) The trajectory of a single PEDOT and PSS chain in the PEDOT-rich-I phase during the annealing cycles. The lower left-hand corner features a representation of the *x* (red), *y* (green) and *z* (blue) axes.

S3- Weight percentage of water to polymer inside the polymer

In the bulk polymer region, the weight percentage of water to polymer inside the phase structures was determined as described in section S1. Figure S4 illustrates the density profiles for PEDOT:PSS. The shaded area in the figure indicates the amount of water in the bulk polymer region. For each phase structure, the wt% denotes the weight percentage of water relative to the polymer in the bulk polymer region.

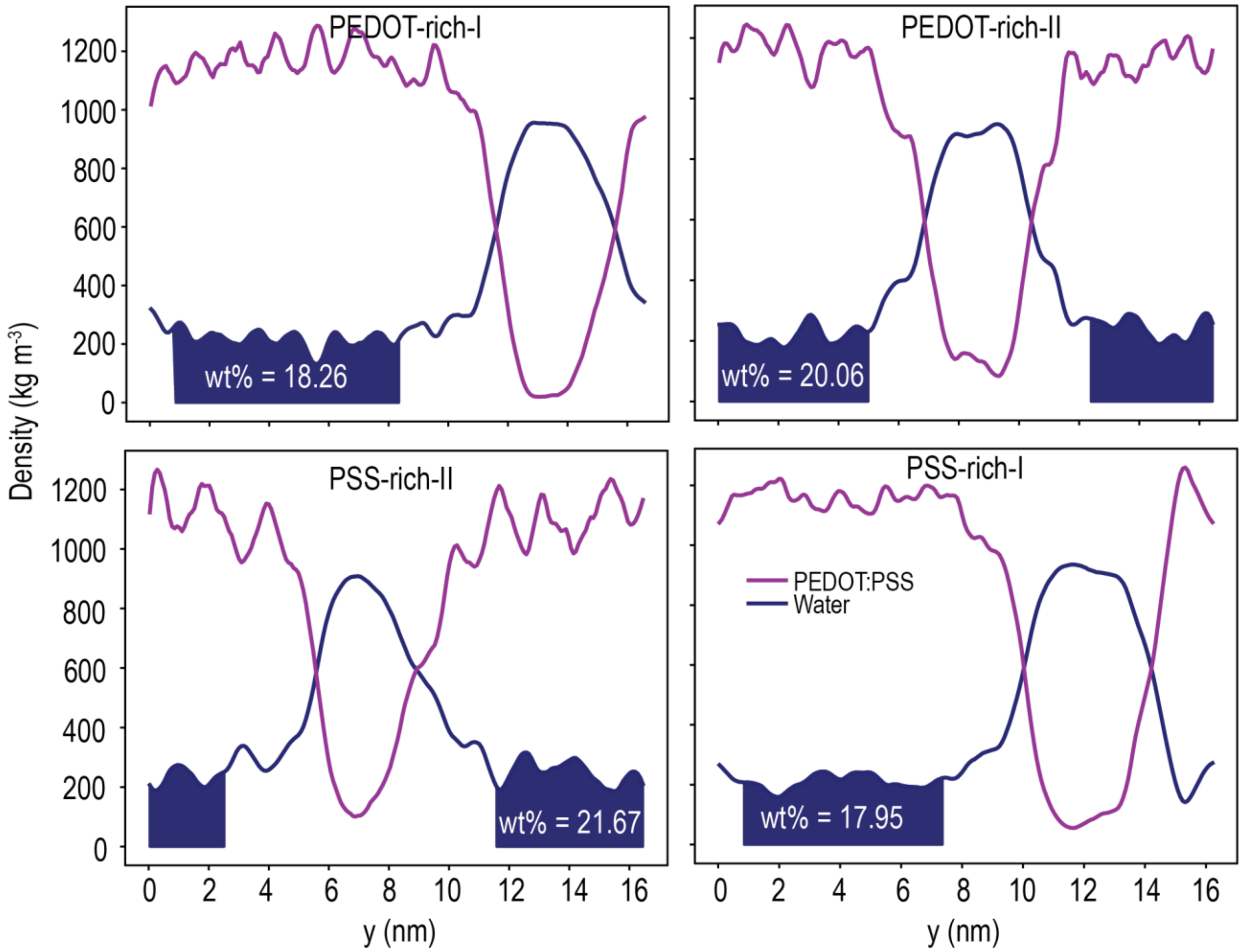

Figure S4 – Density profiles of PEDOT:PSS and water in PEDOT- and PSS-rich phases (calculated during the last 10 ns of the trajectory), illustrating the amount of water (shaded areas) in the bulk polymer regions.

Tables

The annealing schedule employed in a single cycle is shown in Table S1.

Table S1 – Annealing protocol used in the study.

Simulation time (ns)	0	1	3	13	23	24	25
Annealing temperature (K)	310	325	360	360	360	325	310

References

(1) Makki, H.; Troisi, A. Morphology of conducting polymer blends at the interface of conducting and insulating phases: insight from PEDOT:PSS atomistic simulations. *Journal of materials chemistry. C* **2022**, *10* (42), 16126-16137. DOI: 10.1039/d2tc03158b From NLM.

(2) Chaikin, P. M.; Lubensky, T. C. *Principles of Condensed Matter Physics*; Cambridge University Press, 1995. DOI: DOI: 10.1017/CBO9780511813467.

(3) Humphrey, W.; Dalke, A.; Schulten, K. VMD: visual molecular dynamics. *Journal of molecular graphics* **1996**, *14* (1), 33-38.
